# Frailty as a Predictor of Poor Rehabilitation Outcomes among Older Patients Attending a Geriatric Day Hospital Program: An Observational Study

**DOI:** 10.3390/ijerph19106276

**Published:** 2022-05-21

**Authors:** Daniel Andres, Caroline Imhoof, Markus Bürge, Gabi Jakob, Andreas Limacher, Anna K. Stuck

**Affiliations:** 1 BESAS Berner Spitalzentrum für Altersmedizin Siloah, Gümligen, 3073 Bern, Switzerland; andresdaniel91@gmail.com (D.A.); markus.buerge@siloah.ch (M.B.); gabi.jakob@siloah.ch (G.J.); 2 Department of Geriatrics, Inselspital, Bern University Hospital, and University of Bern, 3010 Bern, Switzerland; caroline.imhoof@gmx.ch; 3 CTU, University of Bern, 3012 Bern, Switzerland; andreas.limacher@ctu.unibe.ch

**Keywords:** post-acute care, institutionalization, clinical frailty scale, nursing home

## Abstract

Background: The Geriatric Day Hospital (GDH) is an important outpatient geriatric service, but there are few data on the role of frailty as a potential predictor of poor outcomes in this setting. Methods: Data were analyzed from 499 patients aged ≥ 60 years attending a 12-week GDH program between 2018 and 2021. Frailty status was defined as non-frail (68, 13.6%), mild/moderate frailty (351, 70.3%), and severe frailty (80, 16.0%) based on the Clinical Frailty Scale (CFS). Outcomes were defined as (1) poor outcome (hospital readmission, death, or medical deterioration) during the program and (2) admission to permanent nursing home care upon completion of the program. Multivariate logistic models were used for predictive analyses. Results: The mean age was 80.3 (standard deviation 7.0); 58.3% were women. Overall, 77 patients (15.4%) had a poor outcome, and 48 (9.6%) were admitted to permanent nursing home care. Poor outcome was experienced by none of the non-frail patients (0%), by 49 (14.0%) patients with mild/moderate frailty, and 22 (27.5%) patients with severe frailty (adjusted OR, 2.0; 95% CI 1.3, 3.2; *p* < 0.01). Admission to a permanent nursing home care was experienced by none of the non-frail patients (0%), 20 (5.7%) of those with mild/moderate frailty, and 28 (35.0%) with severe frailty (adjusted OR, 2.9; 95% CI 1.3, 6.3; *p* < 0.01). Conclusions: The CFS is a promising risk predictor of poor outcome and admission to permanent nursing home discharge among older patients attending a GDH program.

## 1. Introduction

A geriatric day hospital (GDH) is an important outpatient geriatric service for older people that focuses on tailored rehabilitation based on a comprehensive geriatric assessment [1]. The primary goal of an outpatient rehabilitation program is to maintain or improve function, prevent adverse medical events, and promote independence so that older adults are able to continue living at home. A clinical risk assessment tool would be helpful to identify people at risk of a poor outcome and permanent nursing home placement. At-risk patients could then be provided with targeted interventions to prevent the occurrence of these outcomes.

Frailty has been shown to be a useful predictive measure to identify vulnerable people in a variety of medical settings [2,3]. For example, among community-dwelling older adults, frailty was shown to be predictive of institutionalization within 10 years of follow-up [4]. However, it is unknown if frailty is a potentially useful measure to identify patients at risk of poor outcomes in a GDH eventually benefitting from targeted interventions.

Evidence of poor outcomes during a GDH program is scarce. Prior studies looked at predictors of 6-month post-discharge outcomes and found that functional discharge outcomes upon the termination of a GHD program were predictive of functional decline 6 months post-discharge [5]. Another study investigated the development of physical performance by a 6 min walk test from admission to discharge in a GDH program [6]. However, we did not find any evidence investigating poor rehabilitation outcomes of a GDH program investigating frailty at admission as a predictor of outcomes.

The purpose of this study was to investigate whether frailty is a potential predictor of a poor outcome and discharge to a nursing home among patients attending a 12-week GDH program.

## 2. Materials and Methods

### 2.1. Study Design

Anonymized data were analyzed for all patients aged 60 years and older who attended the GDH between January 2018 and March 2021 at the BESAS in Bern, Switzerland. Prior to admission, patients were evaluated by the team coordinator and a geriatrician to ensure they met the following criteria: (1) aged 60 years and older, (2) have the potential for functional improvement, (3) able and motivated to participate in rehabilitation sessions, and (4) would benefit from a multidisciplinary team approach. Patients with severe dementia or who resided permanently in a nursing home were not eligible for the GDH program.

The GDH is a 12-week outpatient program that provides an individualized rehabilitation program based on the geriatric assessment performed upon admission to the program. The program components include individual sessions of physical exercise (e.g., gait and balance training, strength training) and cognitive training. Weekly group therapy sessions are also conducted for cognitive training and balance training. On average, a patient receives 2.5 h of therapy per day at the GDH and is instructed to complete a 30 min exercise program at home each day. The number of therapy units is independent of a patient’s functional or frailty status. At the same time, the content of the therapy units is individually adapted (e.g., type and complexity of exercises) according to the patient’s needs and functional status. The GDH staff include a team coordinator, a geriatrician, two nurses, several physiotherapists, an occupational therapist, and a speech therapist. The treating GDH team was not aware of the frailty score of each patient.

General characteristics of the patients, comorbidities, and their social setting were gathered from clinical admission data. Thereby, cognitive impairment and dementia were diagnosed by geriatricians based on standardized cognitive assessments (Montreal Cognitive Assessment). The assessment of functional status was completed upon admission to the GDH. Using information from the assessment, we retrospectively completed the Clinical Frailty Scale (CFS) [7] to determine a frailty category for each patient. The CFS is an ordinal scale ranging from 1–9. A score of 1–4 was considered non-frail, 5–6 mild/moderate frailty, and 7–8 severe frailty, as defined by Rockwood et al. [8]. The score of 9, defining terminally ill patients, did not apply to our patients.

The study was conducted according to the guidelines of the Declaration of Helsinki and approved by the Ethics Committee (Kantonale Ethikkommission Bern, Req-2020-00734, 6 July 2021).

### 2.2. Clinical Outcomes

Based on previous literature that investigated frailty on poor outcomes in geriatric patients [9], we selected and defined the following two clinical outcomes. The occurrence of a poor outcome during the 12-week GDH program and living location after completion of the program were documented for each patient. A poor outcome was defined as one of the following events that resulted in discharge from the GDH program: readmission to acute care, medical deterioration, or death.

We categorized discharge location after completion of the GDH program as (1) continued living at home or (2) permanent nursing home care/death.

### 2.3. Statistical Analyses

Clinical characteristics gathered upon admission to the GDH are presented by absolute and relative frequencies or by mean with standard deviation (sd) for categorical and continuous variables, respectively. Univariate and multivariate regression models adjusted for age (continuous variable), gender (male vs. female), living location upon admission (community living vs. temporary assisted living facility) and cognitive impairment (minor cognitive impairment or dementia vs. no cognitive impairment) were calculated for the CFS (ordinal three-level variable: non-frail, mild/moderate frailty, and severe frailty) and the two outcomes—poor outcome and permanent nursing home discharge. As sensitivity analyses, we calculated receiver operating curves (ROC) for the clinical frailty scale (ordinal variable) regarding a poor outcome and permanent nursing home admission, respectively. As a sensitivity analysis, we calculated univariate and multivariate regression models for patients aged 75 and older (*n* = 391). An a priori decision was made to not perform statistical comparisons among subgroups of patients to avoid type I and II error inflation. Analyses were computed using Stata Version 16.1 (StataCorp LLC, College Station, TX, USA). A *p*-value of <0.05 was considered statistically significant.

## 3. Results

Of the 583 patients admitted to the GDH, 84 patients were excluded from analysis because they did not complete the program due to personal or pandemic-related reasons. Of the remaining 499 patients who were included in the analysis, none were lost to follow-up. The overall mean age was 80.3 years (standard deviation 7.0) ranging from 60 to 96 years, and 58.3% were female. Clinical characteristics are displayed in Table 1. A total of 68 (13.6%) patients were classified as non-frail patients by the CFS, 351 (70.3%) as having mild/moderate frailty, and 80 (16.0%) as having severe frailty. All non-frail patients had a CFS score of 4. None of the patients had a CFS score of 1–3.

During the GDH program, 77 patients (15.4%) had a poor outcome; 33 patients (6.6%) were readmitted to acute care, 42 (8.4%) had a medical deterioration, and 2 (0.4%) died. In an adjusted multivariate analysis, frailty status based on the CFS (non-frail, mild/moderate frailty, and severe frailty) was predictive of a poor outcome (OR, 2.0; 95% CI 1.3, 3.2; *p* < 0.01) (Table 2A). Overall, 48 (9.6%) patients were admitted to permanent nursing home care following discharge from the GDH. In an adjusted multivariate analysis, frailty was predictive of admission to permanent nursing home care (OR, 2.9; 95% CI 1.3, 6.3; *p* < 0.01) (Table 2B). Sensitivity analyses based on the subgroup of patients aged 75 years and older (*n* = 391) gave similar results for both outcomes (supplementary information Table S1A,B).

**Figure 1:**
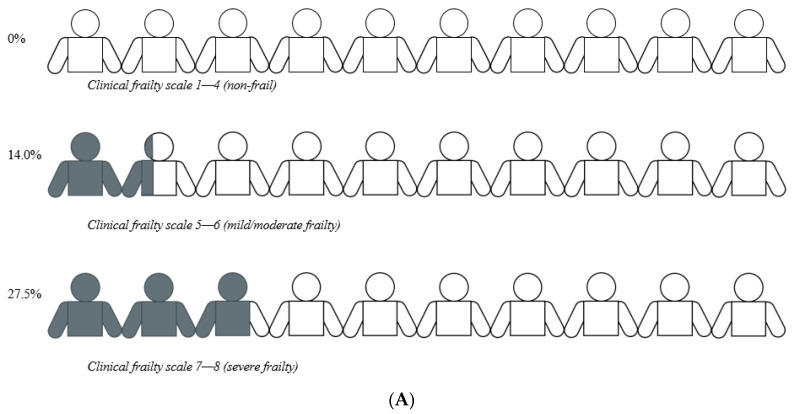

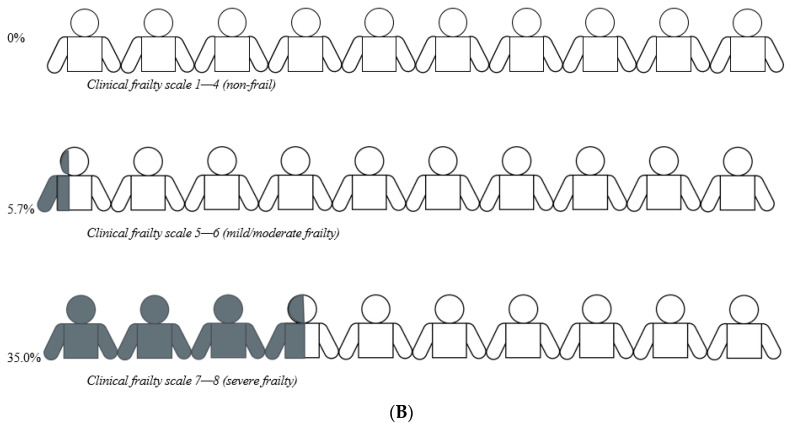
(**A**) Percentages of patients (*n* = 499) who had a poor outcome stratified by their frailty status. Patients with poor outcome are shown in grey. (**B**) Percentages of patients (*n* = 499) admitted to permanent nursing home care stratified by their frailty status. Patients admitted to permanent nursing home care are shown in grey.

There were relevant differences between proportions of patients with a poor outcome and patients without a poor outcome depending on the frailty status. None of the non-frail patients had a poor outcome. Among the mildly/moderately frail patients, 49 (14.0%) had a poor outcome, and among the severely frail patients, 22 (27.5%) had a poor outcome (Figure 1A).

Similarly, there were relevant differences in patients admitted to permanent nursing home care or continued community living, depending on frailty status. Among the mildly/moderately frail patients, 20 (5.7%) were admitted to permanent nursing home care, and among the severely frail patients, 28 (35.0%) were admitted to permanent nursing home care. All of the non-frail patients continued living at home (Figure 1B).

**Figure 2:**
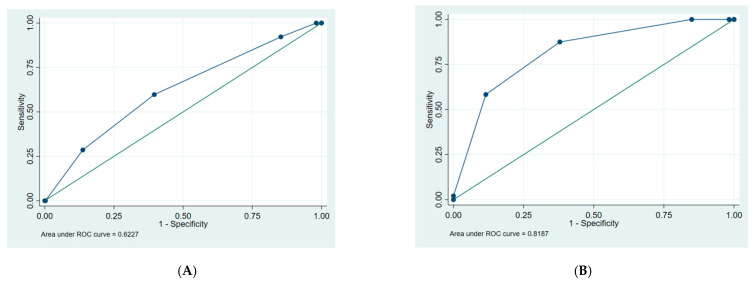
(**A**) Receiver operating curve: Clinical frailty scale and poor outcome. (**B**) Receiver operating curve: Clinical frailty scale and permanent nursing home admission.

Sensitivity analyses showing receiver operating curves of the clinical frailty scale with regard to prediction of a poor outcome and permanent nursing home admission, respectively, are shown in Figure 2A,B.

## 4. Discussion

In this observational study, we observed that the majority of patients admitted to one GDH were mildly/moderately frail based on the CFS classification of frailty. While the level of frailty was predictive of a poor outcome and permanent nursing home admission, the majority of mildly/moderately and severely frail patients did not experience either outcome and were able to continue living at home.

Frailty can be assessed using different approaches, and currently, there is no gold standard measure for assessing frailty. There are two main concepts: physical frailty and cumulative frailty. In the present study, we used the commonly used clinical frailty scale, a judgement-based tool to screen for frailty by summarizing information from a clinical evaluation by a physician or a trained health care professional [8].

Overall, the proportion of frail patients attending the GDH was comparable to what has been reported previously in geriatric inpatient rehabilitation settings. In one such study, 73% of patients were considered frail based on the CFS [9]. At the same time, we observed that few patients experienced a poor outcome or were admitted to permanent nursing home care upon completion of the GDH program. Our results cannot be directly compared to prior evidence due to differences in both outcomes and follow-up times between studies. For example, in two studies of outcomes following GDH, Lim et al. [10] described a hospital readmission rate to acute care of 40% within 1 year of admission to GDH. On the other hand, Luk et al. [5] found a functional decline in 39.2% of patients 6 months after admission.

Our study found that frailty was associated with a poor outcome during the 12-week GDH program and admission to permanent nursing home care. We did not find any other studies conducted in a GDH that investigated frailty as a predictor of these outcomes. In other medical settings, frailty has been shown to be a useful predictive measure to identify vulnerable people [11,12]. For example, frailty was shown to be predictive of future falls in hospitalized patients [13]. Similarly, Stuck et al. found frailty as predictive of non-home discharge and readmission to acute care in an inpatient geriatric rehabilitation setting [9]. Moreover, a recent study showed that several frailty instruments had good sensitivities (but low specificities) in older inpatients for mortality and worsening of basic activities of daily living (BADL) [14]. In turn, some studies in a GDH investigated variables other than frailty. For example, Ono et al. found that patients in a GDH who were older and had higher cognitive function were more likely to be admitted to a long-term nursing home than hospitalized in a dementia ward [15]. Another GDH study found lower functional status upon completion of the program to be predictive of functional decline 6 months after discharge [5].

The strengths of our study include its originality in investigating frailty based on an easy-to-use instrument as a predictor for key clinical outcomes in the setting of a GDH. Therefore, our study addresses an important gap of knowledge in the geriatric field, and the resulting data have important implications for clinical practice and research. Our study sample represents data of a substantial number of older patients (*n* = 499), including old and oldest-old, male and female, patients (age ranges from 60 to 96 years).

However, there are some limitations to our study. First, we investigated data from a single-site GDH program. The GDH model often encompasses different programs and assessments for older patients and is not clearly defined. Therefore, our results are based on a GDH with a specific focus on geriatric rehabilitation measures and may not be representative of other GDH programs. Second, we focused our study on two clinically relevant rehabilitation outcomes (poor outcome and admission to permanent nursing home care). Further study is needed to investigate if there are other outcomes, such as functional outcomes, that are associated with frailty. Third, due to the sample size, we a priori defined a set of clinical variables to calculate a multivariate model. However, there may be other variables that we were not able to include in our predictive models. Fourth, due to the limited sample size, analyses of subgroups were not performed to limit type I and II error. Fifth, we used the CFS, applying the standard cut-offs to assess frailty, since it is one of the most commonly used frailty measures. Our conclusions, though, cannot be extrapolated to other frailty instruments or other cut-offs for defining frailty. Finally, we assessed frailty as a one-point measure upon admission to investigate frailty as a predictor but did not assess frailty over time. Thus, the change of frailty over time is a topic for future study.

Our study has clinical and research implications. Findings from our study demonstrate that frailty based on the CFS is predictive of poor outcomes among GDH, suggesting that frailty may be useful as a risk prediction tool to identify patients at high risk of poor outcomes who might benefit from targeted interventions. At the same time, while the level of frailty was predictive of a poor outcome and permanent nursing home admission, the majority of frail patients did not experience either outcome and were able to continue living at home. Thus, frailty is not a triage instrument, and the determination of who should be included in a GDH program should not be based on frailty status alone.

From a clinical perspective, it would be desirable to identify a single risk predictor to identify patients who are at risk for a poor outcome and permanent nursing home admission. This risk prediction tool should be easy to complete from standard clinical data. The CFS has been shown to be a feasible clinical decision aid in different geriatric settings [16]. However, larger observational studies are needed to confirm the findings of this single-site study, and to investigate if risk stratification using frailty can be used to implement targeted measures to prevent poor outcomes.

## 5. Conclusions

In conclusion, our findings suggest that frailty based on the clinical frailty scale is a promising measure to identify patients at risk of poor outcomes attending a GDH program. Risk stratification based on the CFS combined with targeted interventions may eventually help to prevent poor outcomes in older patients. The findings of this single-site study need confirmation from further studies.

## Figures and Tables

**Table 1:** Clinical characteristics of patients attending the program of the geriatric day hospital (*n* = 499).

General Characteristics	
Age, mean (sd)	80.3 (7.0)
Age ≥ 75 years, *n* (%)	391 (78.4)
Female, *n* (%)	291 (58.3)
Living temporarily in assisted living facility upon admission, *n* (%)	76 (15.2)
Primary indication for admission, *n* (%) - Neurological (e.g., stroke) - Chronic gait and balance disorder - Amputation/prosthesis - Orthopedic reason (surgery and/or acute fracture)	116 (23.3) 273 (54.7) 12 (2.4) 98 (19.6)
Program duration, (days) mean (sd)	85.7 (29.8)
Time to day hospital from current living setting, (minutes) mean (sd)	11.5 (6.9)
Comorbidities	
Cardiovascular disease, *n* (%)	346 (69.3)
Stroke or TIA, *n* (%)	119 (23.8)
Diabetes, *n* (%)	100 (20.0)
Gait and balance disorder, *n* (%)	479 (96.0)
Falls, *n* (%)	197 (39.5)
Depression, *n* (%)	108 (21.6)
Malnutrition, *n* (%)	34 (6.8)
Mild cognitive impairment, *n* (%)	254 (50.9)
Dementia, *n* (%)	37 (7.4)
Vision impairment, *n* (%)	257 (51.5)
Hearing impairment, *n* (%)	171 (34.3)
Functional status and setting	
Dependent on ADL (grooming), *n* (%) ^(a^^)^	235 (47.1)
Dependent on ADL (dressing), *n* (%) ^(b^^)^	174 (34.9)
Home alone, *n* (%)	197 (39.5)
Stairs at home, *n* (%) ^(c^^)^	316 (63.3)
Walking aid, outdoor, *n* (%) ^(d^^)^	384 (77.0)
Walking aid, indoor, *n* (%) ^(b^^)^	282 (56.5)
Frailty	
Clinical Frailty Scale, median (IQR)	5 (5–6)
Frailty status on CFS, *n* (%) - non-frail (CFS score = 4) - mild/moderate frailty (CFS score = 5–6) - severe frailty (CFS score = 7–9)	431 (86.4) 68 (13.6) 351 (70.3) 80 (16.0)
Clinical outcomes	
Poor outcome, *n* (%)	77 (15.4)
Admission to permanent nursing home, *n* (%)	48 (9.6)

*Abbreviations: sd, standard deviation; IQR, interquartile range; CFS, clinical frailty scale; TIA, transitory ischemic attack; ADL, activities of daily living*^(a)^*n* = 572 (*n* = 11 missing) ^(b)^ *n* = 570 (*n* = 13 missing) ^(c)^ *n* = 578 (*n* = 5 missing) ^(d)^ *n* = 559 (*n* = 24 missing).

**Table 2:** (**A**) Clinical characteristics by poor outcome (*n* = 499). (**B**) Clinical characteristics by admission to permanent nursing home care (*n* = 499).

**(A) Clinical Characteristics by Poor Outcome (*n* = 499)**						
	**Regular Completion of GDH (*n* = 422)**	**Poor Outcome (*n* = 77)**	**Unadjusted ** **Odds Ratio (95% CI)**	***p*** **-Value**	**Adjusted ** **Odds Ratio ** **(95% CI) ^(a)^**	***p*** **-Value**
Age, mean (sd) ^(a)^	81.0 (6.8)	80.1 (7.1)	1.0 (0.98, 1.1)	0.35	1.0 (0.98, 1.0)	0.46
Male gender, *n* (%) ^(a)^	170 (40.3)	38 (49.4)	1.4 (0.90, 2.4)	0.14	1.4 (0.86, 2.3)	0.18
Cognitive impairment, *n* (%) ^(a)^	239 (56.6)	50 (64.9)	1.4 (0.85, 2.4)	0.18	1.2 (0.72, 2.0)	0.50
Living temporarily in assisted nursing facility, *n* (%) ^(a)^	64 (15.2)	12 (15.6)	1.0 (0.52, 2.0)	0.93	0.64 (0.30, 1.4)	0.25
Clinical frailty scale, median (IQR) ^(a)^	5 (5–6)	6 (5-7)	2.1 (1.3, 3.3)	<0.01	2.0 (1.3, 3.2)	<0.01
**(B) Clinical Characteristics by Admission to Permanent Nursing Home Care (*n* = 499)**						
	**Continued Community Living (*n* = 451)**	**Admission to Permanent Nursing Home Care (*n* = 48)**	**Unadjusted ** **Odds Ratio (95% CI)**	***p*** **-Value**	**Adjusted Odds Ratio (95% CI) ^(a)^**	***p*** **-Value**
Age, mean (sd) ^(a)^	80.5 (6.8)	78.3 (8.9)	0.96 (0.92, 1.0)	0.04	0.97 (0.92, 1.0)	0.18
Male gender, *n* (%) ^(a)^	192 (42.6)	16 (33.3)	0.67 (0.36, 1.3)	0.22	0.60 (0.26, 1.4)	0.23
Cognitive impairment, *n* (%) ^(a)^	256 (56.8)	33 (68.8)	1.7 (0.89, 3.2)	0.11	1.9 (0.82, 4.6)	0.13
Living temporarily in assisted nursing facility, *n* (%) ^(a)^	36 (8.0)	40 (83.3)	57.6 (25.1, 132)	<0.01	34 (13.9, 83.3)	<0.01
Clinical frailty scale, median (IQR) ^(a)^	5 (5-6)	7 (6–7)	9.5 (5.1, 17.7)	<0.01	2.9 (1.3, 6.3)	<0.01

*Abbreviations: CI, confidence interval; IQR, interquartile range*^(a)^ Variables included in the multivariate logistic model: age (continuous variable), gender (binary variable: male vs. female), cognitive impairment (binary variable: cognitive impairment vs. no cognitive impairment, and frailty status on clinical frailty scale (ordinal three-level variable; non-frail, mild/moderate frailty, severe frailty), living temporarily in assisted living facility upon admission (binary variable: living temporarily in assisted living facility vs. living at home).

## Data Availability

The data presented in this study are available in the article.

## Supplementary Materials

The following supporting information can be downloaded at: https://www.mdpi.com/article/10.3390/ijerph19106276/s1.
