# Supplementary material for: Frailty as a Predictor of Poor Rehabilitation Outcomes among Older Patients Attending a Geriatric Day Hospital Program: An Observational Study
Source: Int J Environ Res Public Health. 2022 May 21;19(10):6276. doi: 10.3390/ijerph19106276 (PMC9140338; doi:10.3390/ijerph19106276)
Supplement: Supplementary file 1 [file ijerph-19-06276-s001.zip › ijerph-1627906-supplementary.pdf]

Supplementary information

For the article entitled:

Frailty as a predictor of poor rehabilitation outcomes among older patients attending a geriatric day hospital program: an observational study

Authors:

Daniel Andres, Caroline Imhoof, Markus Bürge, Gabi Jakob, Andreas Limacher and Anna K. Stuck

April 29, 2022

Table S1. (**A**) Sensitivity analyses: Clinical characteristics by serious adverse events in patients aged 75 and older (n=391). (**B**). Sensitivity analyses: Clinical characteristics by admission to permanent nursing home care in patients aged 75 and older (n=391).

A. Sensitivity analyses: Clinical characteristics by serious adverse events in patients aged 75 and older (n=391)

	Regular completion of GDH (n=422)	Serious adverse event (n=77)	Unadjusted odds ratio (95% CI)	p-value	Adjusted odds ratio (95% CI) a)	p-value
Age, mean (sd) [a)]	83.0 (4.9)	83.2 (5.0)	1.0 (0.96, 1.1)	0.74	1.0 (0.95, 1.1)	0.95
Male gender, n (%) [a)]	131 (39.9)	30 (47.6)	1.4 (0.80, 2.3)	0.26	1.3 (0.73, 2.2)	0.38
Cognitive impairment, n (%) [a)]	198 (60.4)	43 (68.3)	1.4 (0.79, 2.5)	0.24	1.2 (0.68, 2.2)	0.49
Living temporarily in assisted nursing facility, n (%) [a)]	45 (13.7)	9 (14.3)	1.0 (0.48, 2.3)	0.91	0.74 (0.32, 1.7)	0.48
Clinical frailty scale, median (IQR) [a)]	5 (5-6)	6 (5-7)	2.0 (1.2, 3.3)	0.01	2.0 (1.2, 3.5)	0.01

B. Sensitivity analyses: Clinical characteristics by admission to permanent nursing home care in patients aged 75 and older (n=391)

	Continued community-living (n=451)	Admission to permanent nursing home care (n=48)	Unadjusted odds ratio (95% CI)	p-value	Adjusted odds ratio (95% CI) a)	p-value
Age, mean (sd) [a)]	83.0 (4.9)	83.6 (4.7)	1.0 (0.95, 1.1)	0.50	0.99 (0.90, 1.1)	0.79
Male gender, n (%) [a)]	151 (42.1)	10 (31.3)	0.62 (0.29, 1.4)	0.24	0.76 (0.30, 1.9)	0.56
Cognitive impairment, n (%) [a)]	217 (60.5)	24 (75.0)	2.0 (0.86, 4.5)	0.11	1.8 (0.68, 4.82)	0.24
Living temporarily in assisted nursing facility, n (%) [a)]	30 (8.4)	24 (75.0)	32.9 (13.6, 79.6)	<0.01	22.2 (8.6, 57.0)	<0.01
Clinical frailty scale, median (IQR) [a)]	5 (5-6)	6.5 (6-7)	6.3 (3.1, 13.0)	<0.01	2.42 (1.01, 5.8)	0.045

a) Variables included in the multivariate logistic model: age (continuous variable), gender (binary variable: male vs. female), cognitive impairment (binary variable: cognitive impairment vs. no cognitive impairment, and frailty status on clinical frailty scale (ordinal three-level variable; non-frail, mild/moderate frailty, severe frailty), living temporarily in assisted living facility upon admission (binary variable: living temporarily in assisted living facility vs. living at home)
